# Supplementary material for: Extensive length and homology dependent chimerism in pool-packaged AAV libraries
Source: bioRxiv. 2025 Jan 15:2025.01.14.632594. Preprint. [Version 1] doi: 10.1101/2025.01.14.632594 (PMC11761685; doi:10.1101/2025.01.14.632594)
Supplement: Supplement 5 [file NIHPP2025.01.14.632594v1-supplement-5.pdf]

bioRxiv preprint doi: https://doi.org/10.1101/2025.01.14.632594; this version posted January 15, 2025. The copyright holder for this preprint (which was not certified by peer review) is the author/funder, who has granted bioRxiv a license to display the preprint in perpetuity. It is made available under aCC-BY 4.0 International license.

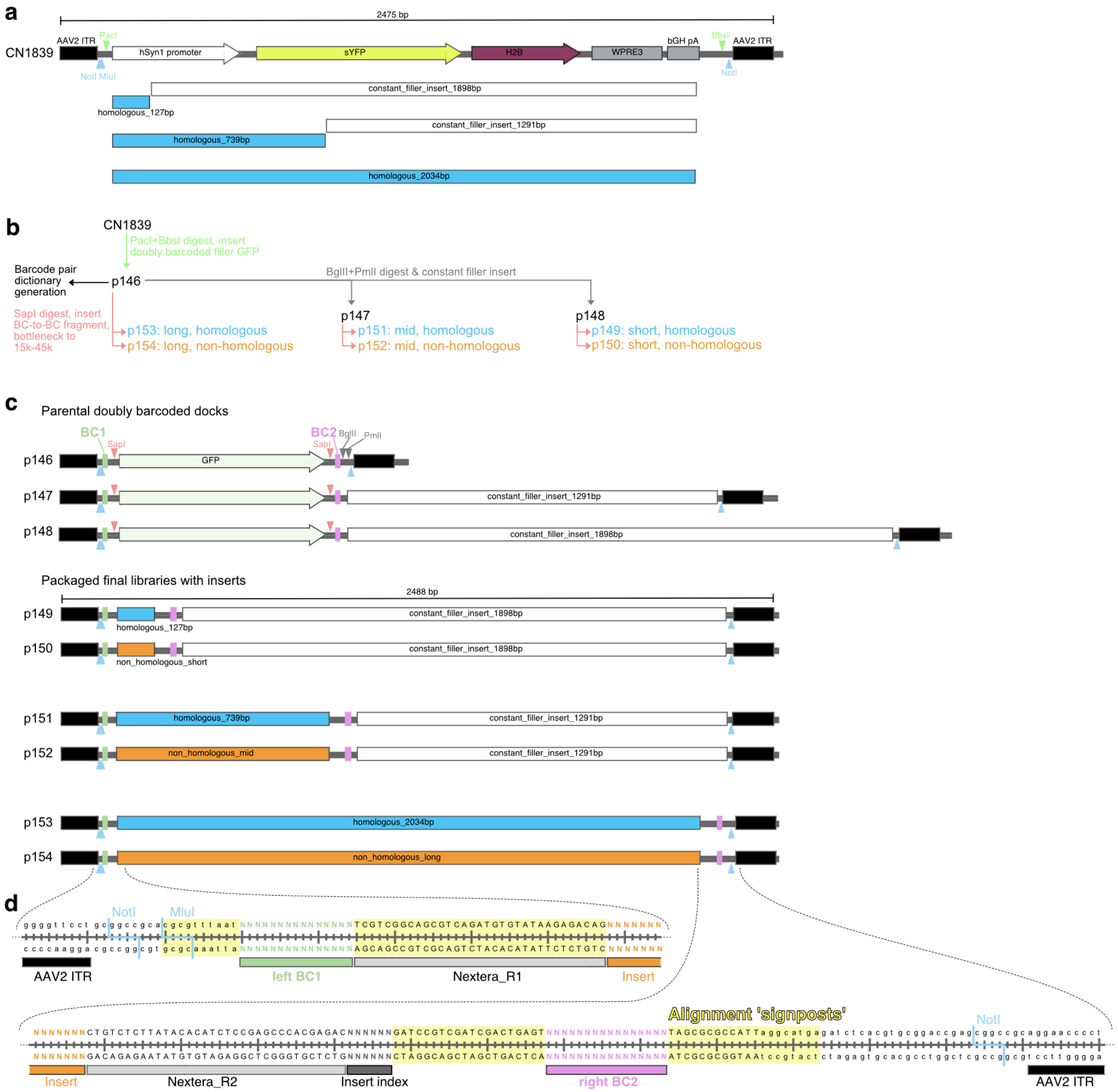

Figure S1: Barcode pairs AAV constructs and cloning strategy.

(a) Schematic of components of plasmid CN1839 (Addgene#163509) between the AAV2 ITRs. Segments under the map highlight constant regions (cloned by PCR) used both as homologous library inserts (blue) and filler sequences to fix the ITR-to-ITR length (white). These segments are shown panel (c) below. Positions of PacI and BbsI restriction sites are marked by green carets, NotI and MluI sites by pale blue carets. **(b)** Molecular cloning scheme used. First complex BC1-BC2 parental dock with GFP stuffer p146 was cloned, and served as template for cloning secondary docks p147 and p148 with filler sequences. Complex library p146 was used as template for barcode dictionary generation. All parental docks were digested with SapI to liberate the GFP, which was replaced by respective internally indexed inserts to generate the final series p149-p154 (which were all bottlenecked to a target of 20k transformants). **(c)** At scale schematics of ITR-to-ITR components of cloned parental and insert-containing libraries. Position of SapI restriction sites are marked by pale red carets, BglII and PmlI sites by grey carets. **(d)** Sequences surrounding the two barcodes highlighting alignment signposts (yellow) used to create local position reference frames around barcodes in the long-read data. The 'Insert index' is shown as Ns, but is fixed and different for each type of insert (not degenerate). Constant Nextera handles between the barcodes (which constitute short homologous regions even in the non-homologous libraries) are shown.

bioRxiv preprint doi: https://doi.org/10.1101/2025.01.14.632594; this version posted January 15, 2025. The copyright holder for this preprint (which was not certified by peer review) is the author/funder, who has granted bioRxiv a license to display the preprint in perpetuity. It is made available under aCC-BY 4.0 International license.

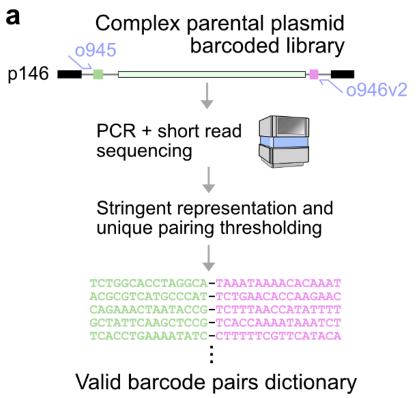

b

Separate BC analysis	Number of barcodes	Read counts	
High-counts left BC1 (reads>300)	609	5374316	
Mid-counts left BC1 (5<reads≤300)	6321002	144288748	
Over-represented right BC2 (ATAACGACTTGTGAGC)	1	3997578	
High-counts right BC2 (reads>300)	420	1308561	
Mid-counts right BC2 (5<reads≤300)	6588391	145519890	

Paired BC analysis	Number of pairs	Read counts	% total reads
All pairs ≥2 reads	10596763	160780560	100.0
↳pairswith mid- or high-counts left BC1	8060914	151770450	94.4
↳pairswith mid- or high-counts right BC2	8152186	149193635	92.8
↳pairswith left BC1 & right BC2 mid- or high-counts	6751379	144208452	89.7
↳leftBC1 >99% reads to unique right BC2	5858954	129544473	80.6
↳rightBC2 >99% reads to unique left BC1	6250085	135931185	84.5
↳rightBC & left BC >99% reads to unique other BC	5586772	122508856	76.2

Final set of valid BC1-BC2 pairs

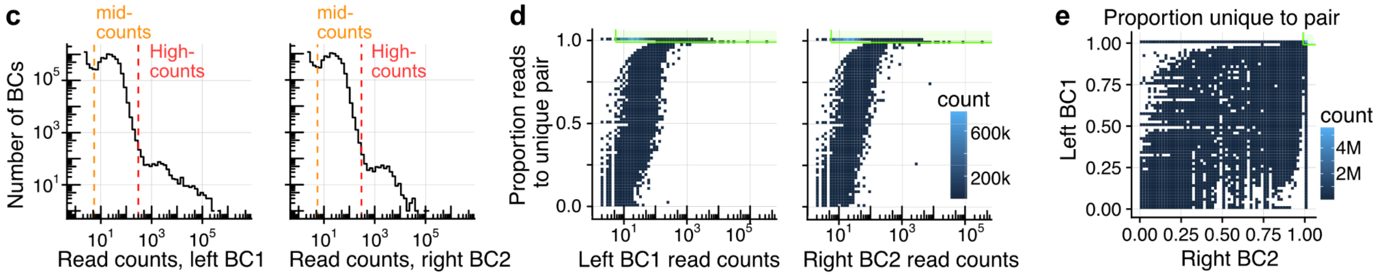

Figure S2: Barcode pairs dictionary generation with paired-end short read sequencing

(a) Schematic of procedure to generate the BC1-BC2 pairs dictionary. Parental plasmid dock p146 was used as template for PCR (primers o945+o946v2) to append Illumina P5 and P7 handles, and sequenced on NS2000 with paired-end sequencing to retrieve barcode pair representation. **(b)** Top: Table of number of barcodes and reads for the different categories shown in panel (c). Bottom: Table of number of barcode pairs, reads, and proportion displaying retention at every filtering step (i.e., both barcodes present in the well-represented set and uniquely paired [>99% reads] with a single other barcode). **(c)** Read count distribution by summing only on respective barcodes (not pairs) for BC1 (left) and BC2 (right). Dashed lines indicate cut-offs used for the mid- and high-counts classes of BCs. **(d)** Two-dimensional distributions showing the proportion of reads to the BC arising from the pair on y-axis (BC1 left panel, BC2 right panel) vs. the total read count to the given pair (x-axis). Retained pairs are within the green boundary (>5 reads and >99% unique proportion). **(e)** Similar to panel (d), but now showing unique pairing proportions for BC1 (y-axis) vs. BC2 (x-axis). Retained pairs are in the top right corner within the green boundaries (>99% uniqueness to both).

bioRxiv preprint doi: https://doi.org/10.1101/2025.01.14.632594; this version posted January 15, 2025. The copyright holder for this preprint (which was not certified by peer review) is the author/funder, who has granted bioRxiv a license to display the preprint in perpetuity. It is made available under aCC-BY 4.0 International license.

a

Filtering step	read counts	proportion total
all reads	2636721	1.000
↳with 4/4 signposts at ≥75% match	913998	0.347
↳coherent strand and positions of signposts	887145	0.336
↳unambiguously demultiplexable	706844	0.268
↳valid left BC1	460901	0.175
↳valid right BC2	470535	0.178
↳both barcodes separately valid	392391	0.149
↳within 1% and 99% BC-to-BC percentile per library	383818	0.146
↳valid barcode pairs	380844	0.144

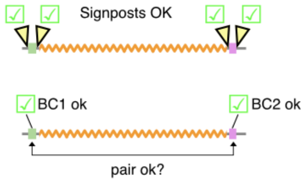

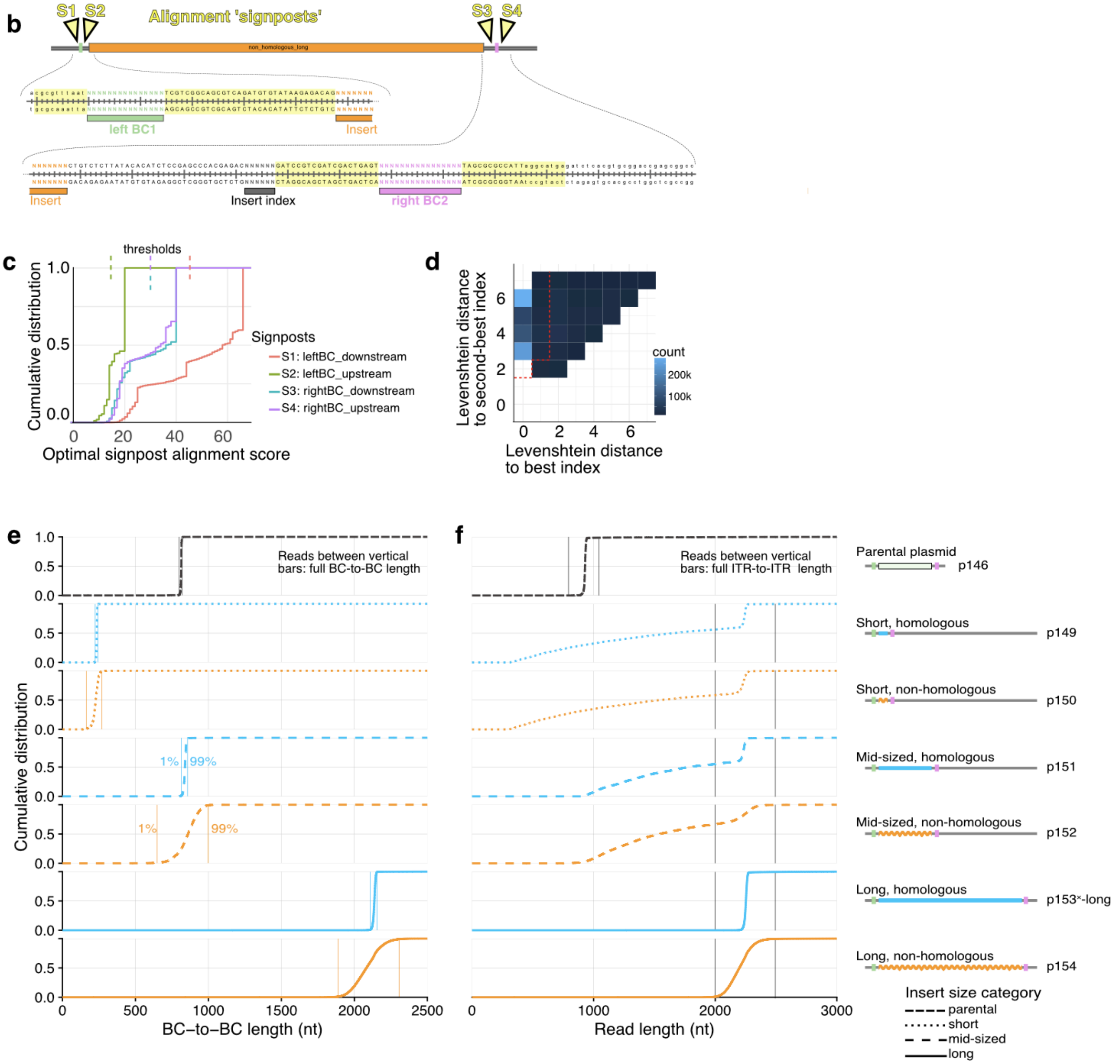

g

Library ID	# reads BC1 & BC2 separately valid	# reads valid BC pair	Fraction valid BC pairs	Quantiles of BC-to-BC length (bp) q01	q10	q90	q99
p146 (parental)	161350	161155	0.999	803.5	809.5	815.5	818.5
p149 (short, homologous)	37618	36710	0.976	226	230.5	237.5	240.5
p150 (short, non-homologous)	49994	49549	0.991	173.5	205.5	248	262.5
p151 (mid-sized, homologous)	9983	9887	0.990	818.5	828.5	847.5	854
p152 (mid-sized, non-homologous)	9592	9538	0.994	690.5	764.55	938.5	981
p153ˣ (long, homologous)	54534	53932	0.989	2114.5	2125.5	2145.5	2151.5
p154 (long, non-homologous)	60747	60073	0.989	1908.5	1970.5	2197	2278.27

Figure S3. Quality control and statistics of long-read data: size-selected plasmid digests
(legend on next page.)

bioRxiv preprint doi: https://doi.org/10.1101/2025.01.14.632594; this version posted January 15, 2025. The copyright holder for this preprint (which was not certified by peer review) is the author/funder, who has granted bioRxiv a license to display the preprint in perpetuity. It is made available under aCC-BY 4.0 International license.

Figure S3. Quality control and statistics of long-read data: size-selected plasmid digests
(a) Table showing long-read counts retention at different filtering steps in our pipeline (schematic at right indicate filter nature, e.g., presence of 4/4 signpost sequences and (separate) exact separate matches to BC1 and BC2 from our dictionary. **(b)** Repeat of **Fig. S1d** re-indicating signpost sequences used to parse the long-read for its insert, BC1, BC2, and insert index. **(c)** Cumulative distribution of alignment scores to the signposts. Thresholds for detection (75% match) are shown as dashed lines at top. **(d)** Two-dimensional histogram showing the Levenshtein distance to the best and second best matches to insert indices. Decision boundary to deem insert demultiplexing as unambiguous is shown by the red dashed line. **(e)** Cumulative distribution of the BC-to-BC length (from middle of both barcodes) as determined by the detected signpost positions from the reads passing signpost quality control steps and with separate exact matches to BC1 and BC2. Line type is related to insert size category (parental, short, mid-sized, long), color to insert class (black: parental, light blue: homologous, orange: non-homologous). Vertical lines indicate 1% and 99% percentiles of the length distributions respectively (used to consider a long-read in the AAV-packaged data as 'full BC-to-BC length'). **(f)** Similar to panel (e), but for total read length. Vertical lines used to consider whether the read is full length or not (parental: 800 to 1050 nt, all other: 2000 to 2500 nt) for analysis in **Table S3**. **(g)** Table of counts of valid reads stratified by demultiplexed libraries (based on insert index), tallying proportion of reads with valid barcode pairs. Quantiles (1%, 10%, 90%, 99%) of the BC-to-BC length across the libraries is also shown.

bioRxiv preprint doi: https://doi.org/10.1101/2025.01.14.632594; this version posted January 15, 2025. The copyright holder for this preprint (which was not certified by peer review) is the author/funder, who has granted bioRxiv a license to display the preprint in perpetuity. It is made available under aCC-BY 4.0 International license.

a

Filtering step	read counts	proportion total
all reads	85306	1.000
↳with 4/4 signposts at >=75% match	11915	0.140
↳coherent strand and positions of signposts	11294	0.132
↳unambiguously demultiplexable	8627	0.101
↳valid left BC1	5843	0.068
↳valid right BC2	6067	0.071
↳both barcodes separately valid	4811	0.056
↳within 1% and 99% BC-to-BC percentile per library	4298	0.050
↳valid barcode pairs	3141	0.037

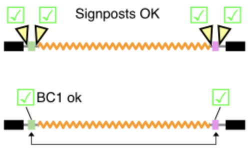

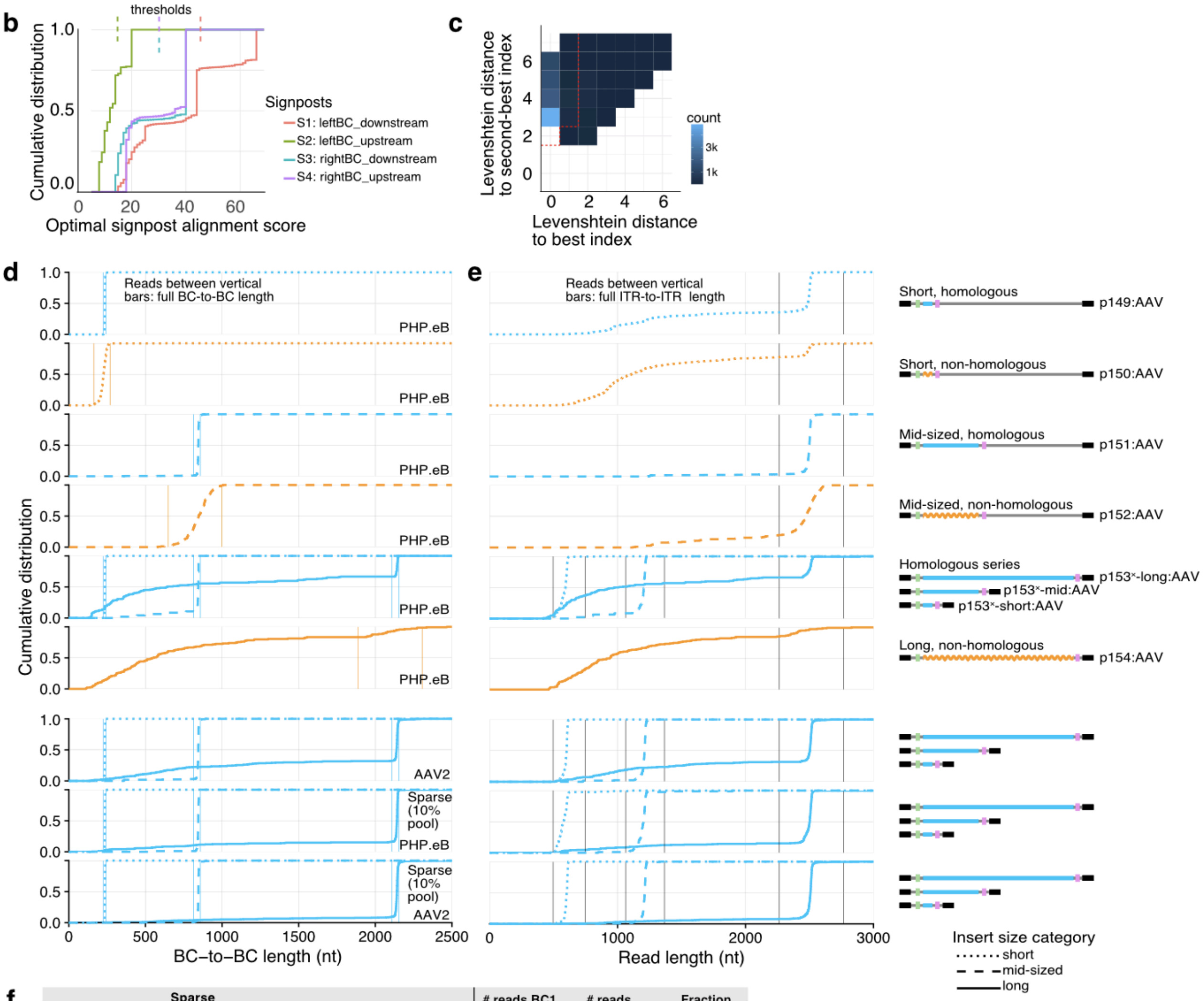

f

Sample ID	serotype	Sparse packaging (10% of pool)	Library ID	# reads BC1 & BC1 valid (separately)	# reads valid BC pair	Fraction valid BC pair
**Full BC-to-BC length reads (within 1%-99% insert length percentiles from plasmid size distributions Fig. S3e)**						
1	PHP.eB	No	p149 (short, homologous)	327	256	0.783
2	PHP.eB	No	p150 (short, non-homologous)	496	470	0.948
3	PHP.eB	No	p151 (mid-sized, homologous)	1444	915	0.634
4	PHP.eB	No	p152 (mid-sized, non-homologous)	165	160	0.970
5	PHP.eB	No	p153ˣ (short, homologous)	259	188	0.726
5	PHP.eB	No	p153ˣ (mid-sized, homologous)	41	18	0.439
5	PHP.eB	No	p153ˣ (long, homologous)	48	19	0.396
6	PHP.eB	No	p154 (long, non-homologous)	21	20	0.952
7	AAV2	No	p153ˣ (short, homologous)	144	95	0.660
7	AAV2	No	p153ˣ (mid-sized, homologous)	155	81	0.523
7	AAV2	No	p153ˣ (long, homologous)	193	96	0.497
8	PHP.eB	Yes	p153ˣ (short, homologous)	120	104	0.867
8	PHP.eB	Yes	p153ˣ (mid-sized, homologous)	83	63	0.759
8	PHP.eB	Yes	p153ˣ (long, homologous)	215	130	0.605
9	AAV2	Yes	p153ˣ (short, homologous)	54	47	0.870
9	AAV2	Yes	p153ˣ (mid-sized, homologous)	136	93	0.684
9	AAV2	Yes	p153ˣ (long, homologous)	397	270	0.680
**Non full BC-to-BC length reads (long insert libraries)**						
5	PHP.eB	No	p153ˣ (long, homologous)	96	10	0.104
6	PHP.eB	No	p154 (long, non-homologous)	117	14	0.120
7	AAV2	No	p153ˣ (long, homologous)	105	7	0.067
8	PHP.eB	Yes	p153ˣ (long, homologous)	43	11	0.256
9	AAV2	Yes	p153ˣ (long, homologous)	65	27	0.415

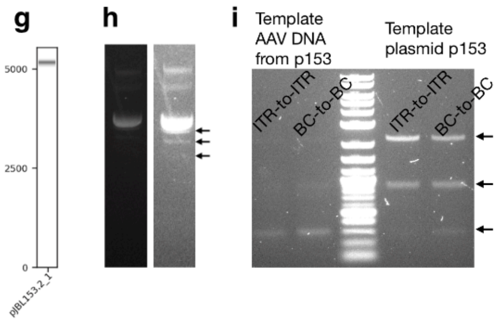

Figure S4. Quality control and statistics of long-read data: direct AAV
(legend on next page)

bioRxiv preprint doi: https://doi.org/10.1101/2025.01.14.632594; this version posted January 15, 2025. The copyright holder for this preprint (which was not certified by peer review) is the author/funder, who has granted bioRxiv a license to display the preprint in perpetuity. It is made available under aCC-BY 4.0 International license.

Figure S4. Quality control and statistics of long-read data: direct AAV

Similar to **Fig. S3** with related modifications. **(a)** Table showing long-read counts retention at different filtering steps in our pipeline (schematic at right indicate filter nature), e.g., presence of 4/4 signpost sequences and (separate) exact matches to BC1 and BC2 from our dictionary. **(b)** Cumulative distribution of alignment scores to the signposts. Thresholds for detection (75% match) are shown as dashed lines at top. **(c)** Two-dimensional histogram showing the Levenshtein distance to the best and second best matches to insert indices. Decision boundary to deem insert demultiplexing as unambiguous is shown by the red dashed line. **(d)** Cumulative distribution of the BC-to-BC length (from middle of both barcodes) as determined by the detected signpost positions from the reads passing signpost quality control steps and with separate exact matches to BC1 and BC2. Each panel comes from a separate AAV packaging sample, and was indexed separately (Plasmidsaurus) for ONT sequencing. For p153$^{\times}$ samples, different inserts were demultiplexed using the internal insert index. Line type is related to insert size category (parental, short, mid-sized, long), color to insert class (black: parental, light blue: homologous, orange: non-homologous). Vertical lines indicate 1% and 99% percentiles from the plasmid libraries (same as **Fig. S3e**) used to call a read ‘full BC-to-BC length’. Note the substantial fraction of reads with shorter than expected lengths for the long insert libraries. **(e)** Similar to panel (d), but for total read length. Vertical lines used to consider whether the read is full ITR-to-ITR or not (p153$^{\times}$-short: 500 to 750 nt, p153$^{\times}$-mid: 1100 to 1400 nt, all others: 2250 to 2750 nt) for analysis of **Table S3**. **(f)** Table of counts of valid reads stratified by demultiplexed libraries (based on insert index), tallying proportion of reads with valid barcode pairs. Quantiles (1%, 10%, 90%, 99%) of the BC-to-BC length across the libraries is also shown. **(g)** Plasmidsaurus (order ID L9RV6B) virtual gel for confirmation of p153, detecting only a single product. **(h)** Agarose gel (low and high contrast) of undigested p153, showing evidence of possible lower molecular weight products (arrows). **(i)** PCR from both AAV template from p153 [serotype PHP.eB, standard condition, sample 5] (left) and plasmid (right) with respectively primers o949+o950 (ITR-to-ITR) and o949+o936 (BC-to-BC). We note that band intensities are probably not representative of species abundance due to possible length-bias of amplification. The three products are of the expected sizes for libraries with short, mid-sized, and long inserts respectively, consistent with their detection in the long-read data.

bioRxiv preprint doi: https://doi.org/10.1101/2025.01.14.632594; this version posted January 15, 2025. The copyright holder for this preprint (which was not certified by peer review) is the author/funder, who has granted bioRxiv a license to display the preprint in perpetuity. It is made available under aCC-BY 4.0 International license.

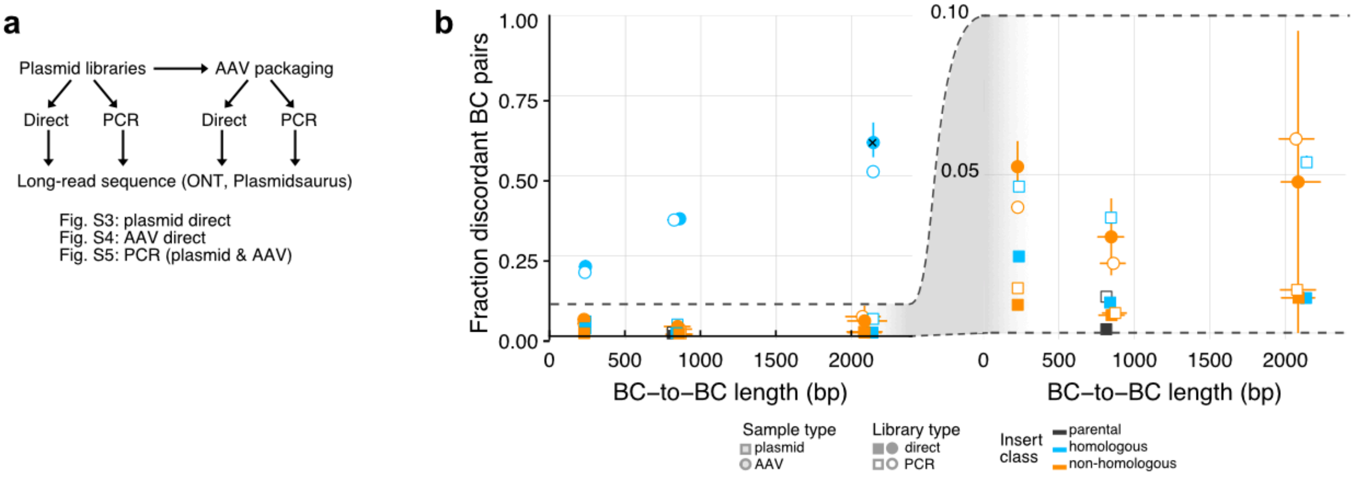

c Statistics filtering, PCR-prep, AAVs template

Filtering step	read counts	proportion total
all reads	1310394	1.000
↳with 4/4 signposts at >=75% match	363371	0.277
↳coherent strand and positions of signposts	270519	0.206
↳unambiguously demultiplexable	243951	0.186
↳valid left BC1	162377	0.124
↳valid right BC2	156519	0.119
↳both barcodes separately valid	123969	0.095
↳within 1% and 99% BC-to-BC percentile per library	108963	0.083
↳valid barcode pairs	87117	0.066

d Statistics filtering, PCR-prep, plasmids template

Filtering step	read counts	proportion total
all reads	414360	1.000
↳with 4/4 signposts at >=75% match	232989	0.562
↳coherent strand and positions of signposts	215315	0.520
↳unambiguously demultiplexable	193279	0.466
↳valid left BC1	131127	0.316
↳valid right BC2	132751	0.320
↳both barcodes separately valid	113955	0.275
↳within 1% and 99% BC-to-BC percentile per library	111898	0.270
↳valid barcode pairs	108675	0.262

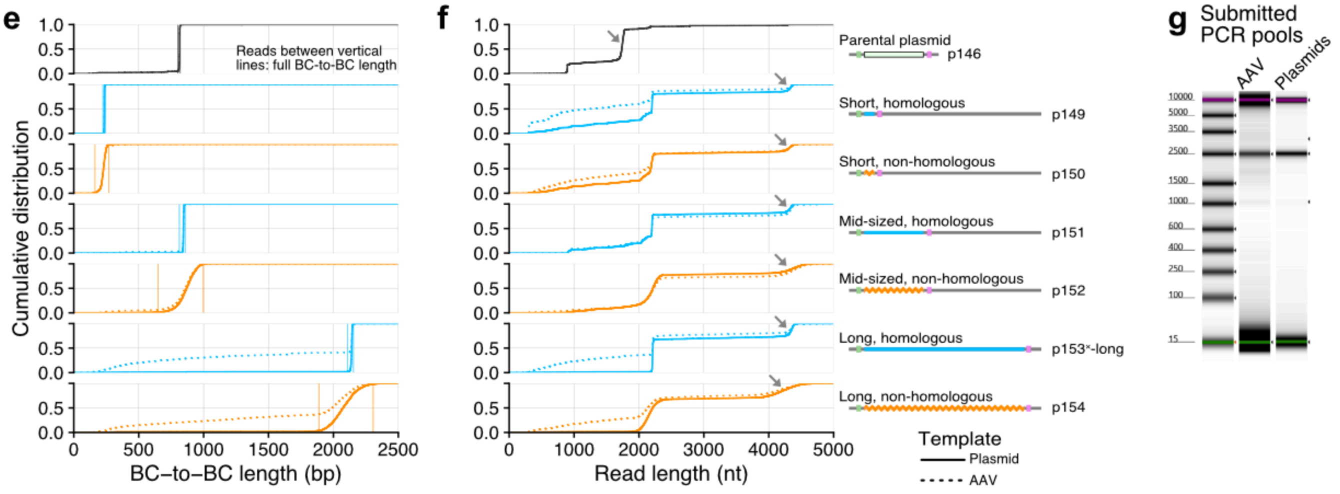

h

	Starting material	Library ID	Direct prep (same data as Fig. S3 & S4) # reads BC1 & BC2 separately valid	# reads valid BC pairs	Fraction valid BC pairs	PCR prep # reads BC1 & BC2 separately valid	# reads valid BC pairs	Fraction valid BC pairs
Only PHP.eB with standard condition (samples 1 to 6)	AAV	p149 (short, homologous)	327	256	0.783	28809	23076	0.801
	AAV	p150 (short, non-homologous)	496	470	0.948	13730	13186	0.960
	AAV	p151 (mid-sized, homologous)	1444	915	0.634	30837	19658	0.637
	AAV	p152 (mid-sized, non-homologous)	165	160	0.970	16528	16165	0.978
	AAV	p153* (long, homologous)	48	19	0.396	6549	3187	0.487
	AAV	p154 (long, non-homologous)	21	20	0.952	9024	8472	0.939
	plasmid	p146 (parental)	161350	161155	0.999	2793	2761	0.989
	plasmid	p149 (short, homologous)	37618	36710	0.976	29043	27703	0.954
	plasmid	p150 (short, non-homologous)	49994	49549	0.991	21427	21124	0.986
	plasmid	p151 (mid-sized, homologous)	9983	9887	0.990	22992	22156	0.964
	plasmid	p152 (mid-sized, non-homologous)	9592	9538	0.994	18860	18741	0.994
	plasmid	p153* (long, homologous)	54534	53932	0.989	9071	8583	0.946
	plasmid	p154 (long, non-homologous)	60747	60073	0.989	7712	7607	0.986
	**Non-full length reads (long insert libraries)**							
	AAV	p153* (long, homologous)	96	10	0.104	4681	283	0.060
	AAV	p154 (long, non-homologous)	117	14	0.120	5577	1296	0.232

Figure S5. Quality control and statistics of long-read data from PCR libraries
(legend on next page)

bioRxiv preprint doi: https://doi.org/10.1101/2025.01.14.632594; this version posted January 15, 2025. The copyright holder for this preprint (which was not certified by peer review) is the author/funder, who has granted bioRxiv a license to display the preprint in perpetuity. It is made available under aCC-BY 4.0 International license.

Figure S5. Quality control and statistics of long-read data from PCR libraries

(a) Flowchart illustrating the different types of libraries considered (starting material: plasmids & AAV packaged DNA), method (PCR-based vs. direct, i.e., PCR-free, corresponding to digestion & size selection for plasmids, and annealing fpr AAVs, prior to end-repair & adapter ligation). PCR libraries were pooled prior to submission and library identity assigned by demultiplexing based on the internal index (see Fig. S1d). **(b)** Similar to Fig. 1c, now also showing the PCR-derived libraries (open symbols). This data is represented as a comparison between direct/PCR libraries in Fig. 1e. **(c)** and **(d)** Table showing long-read counts retention at different filtering steps in our pipeline for PCR libraries prepared from AAV packaged DNA and plasmids respectively. **(e)** Cumulative distribution of the BC-to-BC length (from middle of both barcodes) as determined by the detected signpost positions from the reads passing signpost quality control steps and with separate exact matches to BC1 and BC2. Line type corresponds to template type (full: plasmid, dashed: AAV), color to insert class (black: parental, light blue: homologous, orange: non-homologous). Vertical lines indicate 1% and 99% percentiles of the length distributions respectively from the plasmid digest (and used to consider a long-read in the AAV-packaged data as ‘full BC-to-BC length’). Panels correspond to different insert libraries. Parental inserts from the AAV sample is not shown as it cannot be reliably attributed to a sample (came from residual empty plasmids packaged from all libraries). **(f)** Similar to panel (e), but for the full read length. Grey arrows indicate ‘dimer reads’ (see **Methods**) that were roughly twice the length of the expected full length reads. **(g)** Tapestation (D5000) of pooled PCR-prepared libraries submitted for ONT sequencing, showing predominant expected product (≈2.2 kb), and minor empty product from p146 (≈900 bp), and lack of product >4 kb corresponding to ‘dimer reads’ seen in (f). **(h)** Table of read counts with valid barcode pairs and valid fraction across libraries originating from plasmids or AAV packaged DNA (different rows), and direct (PCR-free) [central columns] vs. PCR-derived libraries [right columns]. The data from the direct libraries is reproduced from Fig. S3 and S4 for plasmid digest and AAVs respectively.
